# Photocatalytic Degradation of Organic Pollutants—Nile Blue, Methylene Blue, and Bentazon Herbicide—Using NiO-ZnO Nanocomposite

**DOI:** 10.3390/nano14050470

**Published:** 2024-03-05

**Authors:** Sadaf Yasmeen, Luca Burratti, Leonardo Duranti, Emanuela Sgreccia, Paolo Prosposito

**Affiliations:** 1 Industrial Engineering Department, University of Rome Tor Vergata, Via del Politecnico 1, 00133 Rome, Italy; sadaf.yasmeen@students.uniroma2.eu (S.Y.); emanuela.sgreccia@uniroma2.it (E.S.); paolo.prosposito@uniroma2.it (P.P.); 2 Department of Sciences, University of Roma Tre, Via della Vasca Navale 79, 00146 Rome, Italy; 3 Department of Chemical Science and Technologies, University of Rome Tor Vergata, Via della Ricerca Scientifica 1, 00133 Rome, Italy; leonardo.duranti@uniroma2.it

**Keywords:** NiO-ZnO nanocomposite, co-precipitation method, photocatalysis, water pollutants, herbicide, dyes

## Abstract

Water pollution poses a significant threat to both human health and ecosystem integrity. Chemical pollutants such as dyes and pesticides affect the water quality and endanger aquatic life. Among the methods for water purification from organic pollutants, photodegradation is certainly a valid technique to decrease such contaminants. In this work, pristine NiO, ZnO, and NiO-ZnO photocatalysts were synthesized by the homogeneous co-precipitation method. X-ray diffraction confirms the formation of a photocatalyst consisting of ZnO (Hexagonal) and NiO (Cubic) structures. The crystalline size was calculated by the Scherrer formula, which is 19 nm for the NiO-ZnO photocatalyst. The band gap measurements of the prepared samples were obtained using the Tauc Plot, equation which is 2.93 eV, 3.35 eV and 2.63 eV for NiO, ZnO, and NiO-ZnO photocatalysts, respectively. The photocatalytic performance of NiO-ZnO nanocomposite was evaluated through the degradation of Methylene Blue and Nile Blue dyes under sunlight, and Bentazon herbicide under a UV light. Photocatalyst degradation efficiency was 95% and 97% for Methylene Blue and Nile Blue in 220 min under sunlight while a degradation of 70% for Bentazon after 100 min under UV light source was found.

## 1. Introduction

Currently, water pollution is a global issue due to its harmful effects on water species, human beings as well as animals. The release of herbicides from intensive agriculture and organic dyes from industries into freshwater reservoirs without any pretreatment has potential health effects on living beings [1,2].

Nowadays, modern agriculture uses various herbicides for the better growth of agriculture, controlling different kinds of pests and improving the food [3]. Similarly, various synthetic dyes are used in medical laboratories and industries like paint, textiles, food, and printing. The excess release of these herbicides and dyes is highly toxic for the water environment, soil fertility, aquatic creatures, and biological ecosystems. In this regard, the World Health Organization (WHO) sets the threshold levels of herbicides in drinking water at approximately 30 µg/L [4,5].

To overcome this issue, several methods have been employed to remove organic dyes and herbicides, such as coagulation, sedimentation, reverse osmosis, biological and chemical reactions, and photocatalytic activity [6]. Each method has its own advantages and limitations. In recent years, among these methods, semiconductor-mediated solar photocatalysis has been considered an efficient technique for the removal of these organic pollutants as it is an eco-friendly and sustainable approach to degrading the toxic pollutants into nontoxic molecules [7].

A literature review revealed that different semiconductor photocatalysts have been used for the degradation of dyes and herbicides. Semiconductors can be divided into two classes; n-type semiconductors, such as ZnO, CeO_2_, TiO_2_, SnO_2_, WO_3_, etc. [8,9,10,11,12,13] and p-type semiconductors that include NiO, Co_3_O_4_, Mn_3_O_4_, etc. [14,15,16]. Using a single metal oxide, semiconductor photocatalysts high recombination rate and poor charge carrier mobility limit the photocatalytic activity. Several techniques have been adopted, such as the mixing of two or more semiconductors [4], single doping [17], dual doping [18], and co-doping [19], to improve their charge transport properties and prevent electron-hole recombination. Metal oxides can be synthesized by different approaches, such as precipitation [20] or co-precipitation in case of two or more metal precursors [21,22], precipitation in the presence of chelating agents [23,24] or even more complex methods [25,26]. Among these approaches, the co-precipitation method ensures an easy, fast, and industrially scalable synthesis. In addition, the obtained materials have gained much attention due to their high efficiency in absorption, electron hole pair generation, and high efficiency when used for wastewater treatment and other applications [27]. In recent years, several studies have been reported on the removal of industrial dyes and herbicides using p-n heterojunction semiconductor photocatalysts such as ZnO/CdO, CuO/TiO_2_, ZnO/MgO, ZnO/WO_3_, NiO/ZnO [28,29,30,31,32,33] for the removal of synthetic dyes and Fe_2_O_3_-TiO_2_ [1], ZnO/CuO [34], and Ag/TiO_2_ [35] for the removal of different herbicides.

Although NiO-ZnO nanocomposite has been widely investigated for the degradation of different synthetic dyes, to the best of our knowledge, the activity of such a photocatalyst towards the degradation of Nile Blue and Bentazon has not been reported. In this work, pristine ZnO and NiO nanoparticles, along with a NiO-ZnO photocatalyst (NZP), were prepared by the co-precipitation method. The structural, morphological, photocatalytic, and optical properties were studied using X-ray diffraction (XRD), Scanning electron microscopy (SEM), Ultraviolet and visible (UV-Vis) spectroscopy, Fourier transform infrared (FTIR), and Raman spectroscopies. The degradation efficiency of the prepared photocatalyst was evaluated on Methylene Blue (MB) and Nile Blue (NB) dyes (cationic), and Bentazon (BZ) herbicides (anionic).

## 2. Materials and Methods

### 2.1. Chemicals

Nickel nitrate hexahydrate [Ni(NO_3_)_2_·6H_2_O, CAS No: 13478-00-7, purity 99%, crystals] and zinc nitrate hexahydrate [Zn(NO_3_)_2_·6H_2_O, CAS No: 10196-18-6, purity 98%, crystals] were used as precursors for the synthesis of NiO, ZnO and NZP. Sodium hydroxide (NaOH, CAS No: 1310-73-2, purity 97%, pallets) was employed as a precipitating agent. Hydrochloric acid (HCl, CAS No: 7647-01-0, concentration 37%, density 1.2 g/mL) was used for changing the pH. For the photocatalytic activity Methylene Blue (C_16_H_18_ClN_3_S, CAS No: 61-73-4, dye content ≥ 82%, powder), Nile Blue (C_40_H_40_N_6_O_6_S, CAS No: 3625-57-8, dye content ≥ 75%, powder) and Bentazon (C_10_H_12_N_2_O_3_S, CAS No: 25057-89-0, purity ≥ 98%, powder) were used as pollutants in deionized water. All the reagents were purchased from Merck (Darmstadt, Germany) and used as received without any further refinement procedures.

### 2.2. Synthesis of Pristine NiO, ZnO and NiO-ZnO Photocatalyst

**Figure 1:**
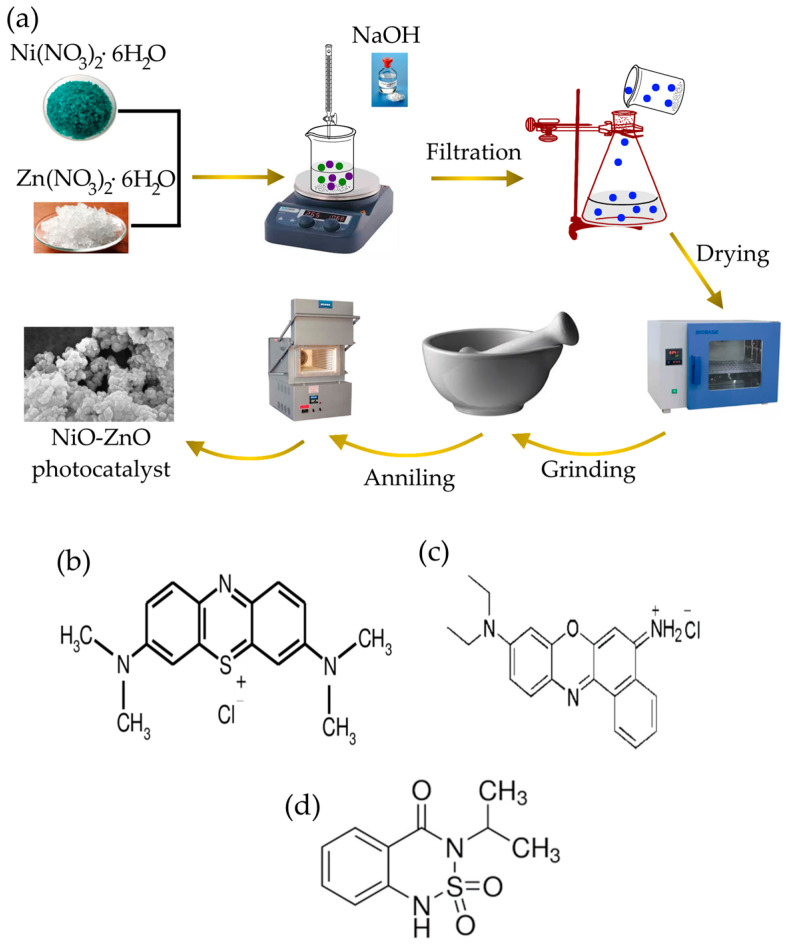
(**a**) Schematic representation of the synthesis process of NZP photocatalyst. Structure formula of (**b**) Methylene Blue dye, (**c**) Nile Blue dye and (**d**) Bentazon herbicide.

ZnO, NiO nanoparticles, and NZP were prepared using the homogeneous co-precipitation method. For the synthesis of NiO and ZnO nanoparticles, nickel nitrate hexahydrate (2.9079 g) and zinc nitrate hexahydrate (2.9748 g) were mixed in two separate beakers in 50 mL deionized water and magnetically stirred for 1 h. After 1 h, NaOH solution (1 M) was added dropwise to each solution until pH 9 was reached, and then the solutions were stirred for 3 h. The greenish precipitate for NiO and the white precipitate for ZnO started to form. The obtained precipitates were washed to remove impurities with distilled water and then filtered. The resulting products were dried in an oven at 60 °C for 12 h. The synthesis of NZP was based on a similar procedure, starting with a solution with a Zn:Ni mole ratio of 1:1 in 100 mL deionized water with the amount of nickel salt 2.9079 g and zinc salt 2.9748 g. Finally, dried NiO, ZnO, and NZP were grinded to obtain fine powders and then annealed at 600 °C for 2 h. The synthesis process is schematically summarized in Figure 1a.

### 2.3. Photocatalytic Test

The photocatalytic degradation efficiency of the prepared NZP was investigated using Methylene Blue (MB) and Nile Blue (NB) as cationic dyes and Bentazon (BZ) as an herbicide pollutant. In the photocatalytic test, 30 mg of photocatalyst powder was added separately to 60 mL of a water solution of MB, NB, and BZ at a fixed concentration (5 ppm). For the adsorption–desorption equilibrium of dyes and herbicides on the surface of nanocomposite, the pollutant solutions were stirred for 1 h in the dark. The dye solutions containing NZP were exposed to solar light irradiation. In the case of BZ, the experiment was carried out under a mercury lamp (300 W) (Oriel Instruments, Newport, CA, USA), selecting the wavelength range of 220–260 with a dichroic mirror in a dark room. The pollutant solution was placed 20 cm from the lamp. The light intensity of irradiation was measured by a power meter (Thorlabs, Newton, NJ, USA, model PM100D) at wavelength 240 nm, which was about 7.8 mW during all the experiments. After regular intervals of 20 min, 2 mL of solution was taken and analyzed using a double-beam UV-Vis spectrophotometer.

The percentage degradation efficiency of the photocatalyst was calculated using the following formula [36]:

(1) $$\%\;Degradation=\left(C_{0}-C_{t}\right)/C_{0}*100$$

with *C*_0_ and *C_t_* the concentration of pollutants before and after irradiation, respectively. The pH was optimized for BZ, evaluating the degradation efficiency. Three values of pH were explored: 5, 7 and 9; the values were reached using NaOH (1 M) or HCl (1 M). For the three pH values the degradation efficiency was 75%, 70% and 71% for pH 5, 7 and 9, respectively, as reported in Figure S1 of the supporting information. Due to the almost constant degradation efficiency values, for all the activities the pH was set at 7.

The self-degradation of pure organic compounds in water solution was analyzed by exposing MB and NB (concentration 5 ppm) under sunlight and BZ (5 ppm) under UV light source.

The structure of dyes and herbicide are shown in Figure 1.

### 2.4. Reusability of the NZP

The reusability of the photocatalyst was evaluated by repeating the photodegradation process for the BZ under the same reaction conditions. After completing each cycle of degradation, the photocatalyst was washed with deionized water and separated by centrifuging the samples for 10 min at 3000 rpm with a centrifuge (Thermo Fisher, Waltham, MA, USA, Megafuge 8) to collect all of the powder. After, the powder was dried for about 1 h in an oven at 70 °C and it was used again for the next cycle. Figure S5 shows the degradation efficiency of the NiO-ZnO photocatalyst for the three cycles.

### 2.5. Instrumentation

A Philips X-Pert Pro 500 (Amsterdam, The Netherlands) diffractometer X-ray diffraction (XRD) on NiO, ZnO, and NiO-ZnO powders was performed using Cu Kα radiation (*λ* = 1.54056 Å) in the 30–90° 2θ range, with 4 s counting time and 0.02° step size. The morphology of the samples was investigated using a Zeiss Leo SUPRA™ 35 (Oberkochen, Germany) field emission scanning electron microscope (FE-SEM). Elemental Analysis was carried out using the energy-dispersive X-ray (EDX) spectrometer. The Fourier Transform Infrared spectrophotometer (Jasco FT/IR-4X, Victoria, BC, Canada) was used to determine the functional groups. Raman data was collected using ATR8300 Raman using integral time 2000 ms and laser power 25 mW. The Optical and photocatalytic measurements were measured using a double beam UV-Vis spectrophotometer (PerkinElmer UV/VIS/NIR spectrometer Lambda 750, Shelton, CT, USA).

## 3. Results and Discussion

### 3.1. X-ray Diffraction

**Figure 2:**
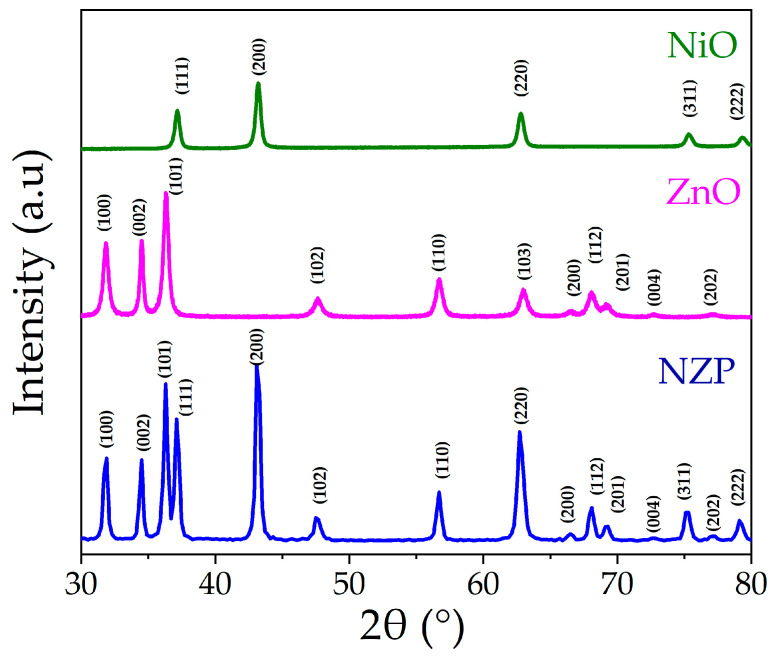
XRD spectra of NiO, ZnO, and NZP.

The crystal structure of the grown samples was determined using X-ray diffraction. The patterns of pristine NiO, ZnO, and NZP photocatalysts are shown in Figure 2.

Both NiO and ZnO patterns showed pure-phase samples, with peaks positions and relative intensity that closely match the reference cards of NiO rock-salt structure (JCPDS 47-1049) and ZnO hexagonal structure (JCPDS 36-1451), respectively. The diffraction peaks of both NiO and ZnO are visible in the NZP nanocomposite diffractogram; no extra peaks belonging to secondary phases are observable, indicating that NZP is only made up of NiO and ZnO. The Scherrer equation is used to determine the crystallite size “D” of the grown samples, and it can be written as [37]:

(2) $$D=\frac{k\lambda }{\beta_{hkl}*cos\theta }$$

where *k* is a constant = 0.9, *λ* is the used Cu Kα radiation wavelength = 1.5406 Å, *β* = full width at half maximum of the peak and *θ* is Bragg angle [38]. The higher peaks of intensity of NZP with lower full width of half maxima shows the higher crystallinity of the photocatalyst as compared to the pristine NiO and ZnO as shown in Figure 2. The average crystallite size for NiO, ZNO, and NZP were 20 nm, 17 nm and 19 nm, respectively. The crystallite size and other XRD structural parameters such as lattice constants (a, c), unit cell volume (v), d-spacing (d), dislocation density (ρ) and strain (ε) [37,39] were calculated and listed in Table 1.

### 3.2. SEM Analysis and Energy Dispersive X-ray Spectroscopy

**Figure 3:**
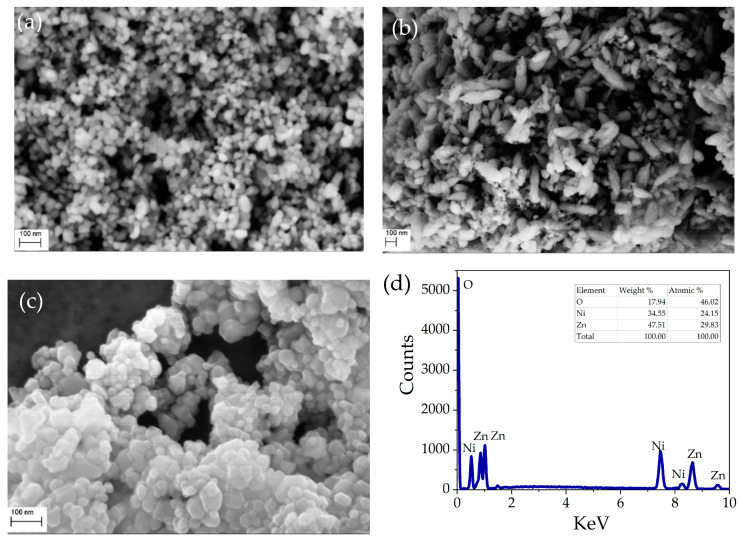
SEM images of: (**a**) the pristine NiO, (**b**) pristine ZnO, and (**c**) NZP; (**d**) EDS spectrum and elemental composition of NZP.

The surface morphology and chemical composition of pristine NiO, ZnO, and NZP were carried out using SEM analysis. The obtained SEM images showed that all the grown samples have nano-sized particles. Figure 3a,b reveals that ZnO nanoparticles have a rice-like structure and NiO nanoparticles have a spherical morphology with a non-homogeneous distribution. The SEM image and elemental composition of the NiO-ZnO photocatalyst are shown in Figure 3c,d. The NZP has uniform and round-shaped nanoparticles. From EDX characterization, the atomic percentage values of Ni, Zn, and O are reported in the inset of Figure 3d, indicating the presence of Nickel, Zinc, and oxygen with atomic percentages of 24%, 29%, and 46%, respectively.

### 3.3. FTIR Analysis

**Figure 4:**
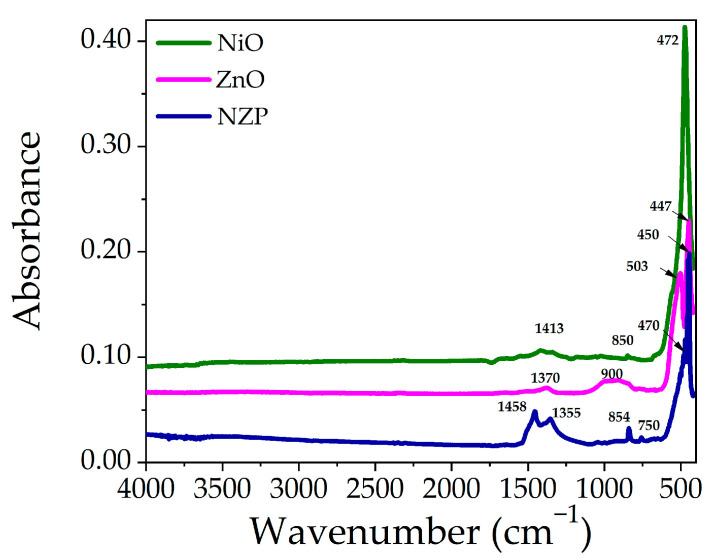
FTIR Spectra of pristine NiO, ZnO, and NZP in the range of 400–4000 cm^−1^.

The Fourier-transform infrared spectroscopy (FTIR) technique is used to study the major functional groups and their vibrational frequencies present in grown samples. The FTIR spectra of Pristine NiO, ZnO, and NZP are displayed in Figure 4. In the low wavenumber region (400–850 cm^−1^), the peaks are related to metal-oxygen (M-O, M = Ni, Zn) and metal-hydroxide (M-OH) bonds [37,40]. The absorption peak at 472 cm^−1^ corresponds to the M-O vibrational mode due to the Ni-O stretching vibrations and the peaks at 447 and 503 cm^−1^ are related to ZnO stretching vibrations, while vibrations in NZP (NiO = 470 cm^−1^, ZnO = 450 cm^−1^) [41]. The stretching vibrations of NiO and ZnO in NZP confirm the formation of a photocatalyst [21,42]. The low-intensity peaks at 850 to 900 cm^−1^ are attributed to tetrahedral Zn^2+^ ions [43]. The peaks at 1300–1460 cm^−1^ are due to the presence of NO_3_, which might not be removed well during the washing process [38].

### 3.4. Optical Analysis

**Figure 5:**
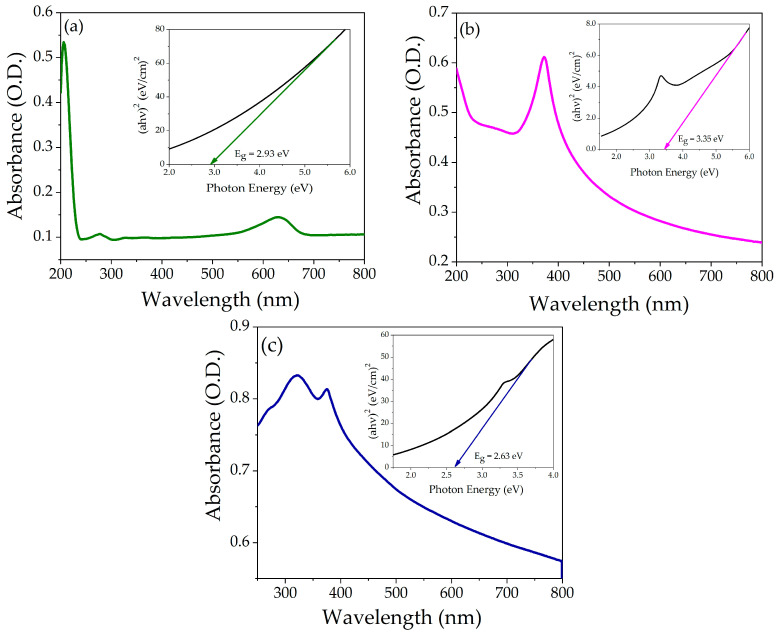
Absorption spectra as a function of wavelength (**a**) NiO, (**b**) ZnO, and (**c**) NZP, the insert shows Tauc’s plot of energy band gap.

The powder of each compound was suspended in water (concentration 5 ppm) separately, and the UV-Vis absorption spectra were recorded. Figure 5a–c shows the absorption spectra of pristine NiO, ZnO, and NZP. The absorption peaks of NiO and ZnO were observed at 279 nm and 370 nm, respectively. In the NiO-ZnO photocatalyst, the absorption peaks were centered at 320 nm and 376 nm, respectively, attributed to NiO and ZnO, which confirms the coexistence of two oxides in a single matrix. The shift in the absorption spectra of the photocatalyst might be due to the incorporation of Zn^2+^ ions into the NiO lattice. The optical energy band gaps of ZnO, NiO, and NZP were analyzed using the Tauc plot equation, which gives the correlation between the incident photon energy (hʋ) and absorption coefficient (α), as shown in Figure 5a–c (insets) [44]. The calculated energy band gaps (Eg) for NiO, ZnO, and NZP were 2.93 eV, 3.35 eV, and 2.63 eV, respectively. These measured values of band gap energy are well consistent with the literature [45,46,47]. The value of Eg of NZP is in the visible region, suggesting that it can enhance photocatalytic activity under sunlight.

### 3.5. Raman Analysis

**Figure 6:**
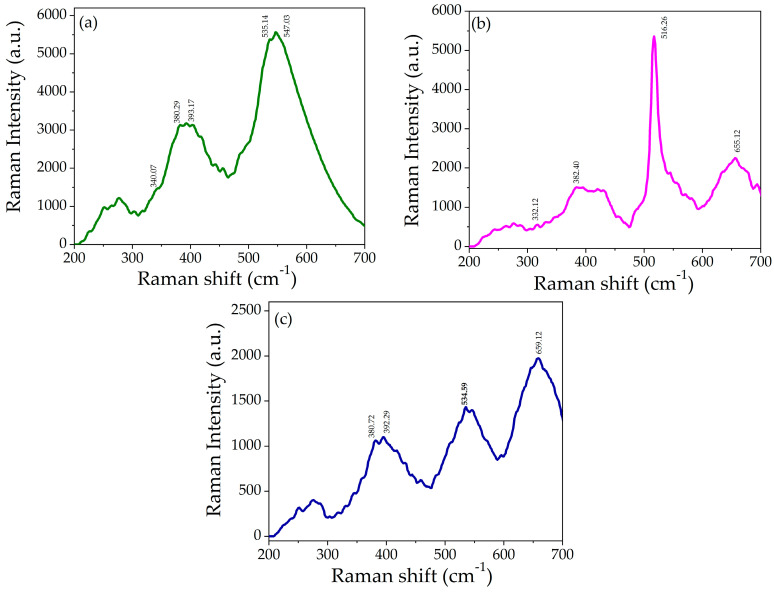
Raman spectra of (**a**) NiO, (**b**) ZnO, and (**c**) NZP.

To study the structural and vibrational properties of prepared samples, the Raman spectroscopy technique was employed. Figure 6a–c shows the Raman spectra of ZnO, NiO nanoparticles and NZP in the spectral range of 200–700 cm^−1^.

The Raman spectrum of NiO showed a low-intensity band at 340.07 cm^−1^, a medium-intensity band at 380.29 cm^−1^ and 393.17 cm^−1^, and a high-intensity band at 535.14 cm^−1^ and 547.03 cm^−1^, as shown in Figure 6a, and they are attributed to the active modes of cubic NiO, one-phonon (1P), one-phonon (TO), and one-phonon (LO), respectively [37,48,49].

The Raman spectrum of ZnO is shown in Figure 6b. It exhibits weak scattering peaks at 332.12 cm^−1^ and 382.20 cm^−1^ assigned to E2 (low) and E2 (high) associated with the motion of oxygen atoms in the lattice and confirmed the wurtzite crystal structure of ZnO [50]. The strong and sharp peaks at 516.26 cm^−1^ correspond to A1 (LO), and the weak peak at 655.12 cm^−1^ is an acoustic overtone with A1 symmetry, which confirmed the formation of ZnO nanoparticles [4,51]. In the Raman spectra of NZP, the NiO phase appeared at 392.29 cm^−1^ and 534.59 cm^−1^ while the ZnO phase appeared at 380.72 cm^−1^ and 659.12 cm^−1^, which confirms the formation of the NiO-ZnO photocatalyst. The optical phonon modes of NiO and ZnO in NZP confirmed the co-existence of two phases in a single matrix. There is a slight shift in the peaks in the spectrum of NZP as shown in Figure 6c, that might be due to phonon confinement, defects (oxygen deficiency, surface impurities), and structural disorder [41,48].

### 3.6. Photocatalytic Activity

The photocatalytic activity of NZP was examined for MB, NB dyes under sunlight and for Bentazon herbicide under UV light at fixed concentrations of 5 ppm of contaminant at different time intervals. The absorption spectra of dyes and herbicide were measured with UV-Vis spectroscopy.

#### 3.6.1. Degradation of Methylene Blue and Nile Blue Dyes

**Figure 7:**
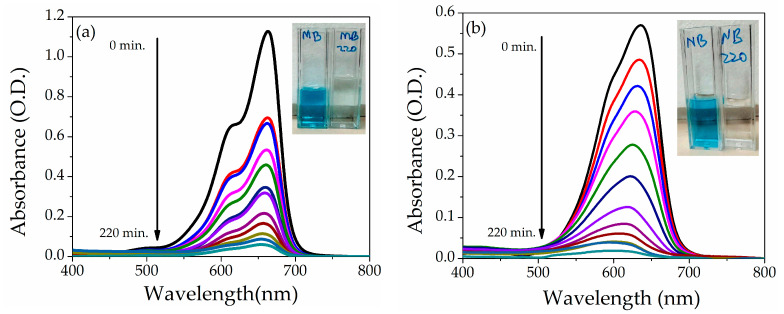
Absorption spectra of (**a**) MB and (**b**) NB at 5 ppm dye concentration in the presence of NZP. Insets show pictures of the cuvettes before and after sunlight irradiation of 220 min.

The photocatalytic activity of NZP was investigated using the two cationic dyes Methylene Blue and Nile Blue at fixed concentration (5 ppm) under the natural sunlight. The maximum absorption peak of MB and NB is observed at ʎ = 664 nm and ʎ = 634 nm, respectively. The photodegradation (absorbance) under direct sunlight of MB and NB dyes in the presence of NZP for various time intervals from 0 to 220 min is shown in Figure 7a,b. An evident decrease of the absorption peak as a function of time can be appreciated. In addition, after 220 min, visual degradation is reported in the inset of Figure 7a,b for MB and NB dyes before and after degradation.

**Figure 8:**
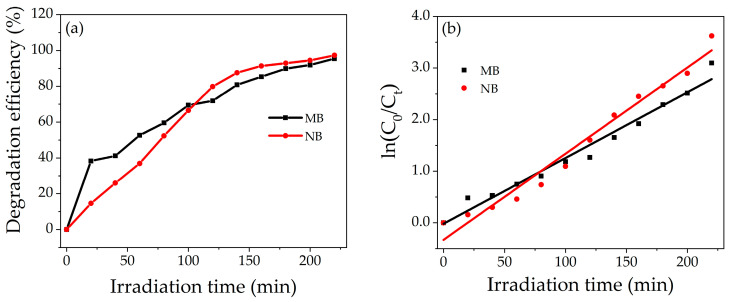
(**a**) Percentage degradation Efficiency of NZP for MB and NB (**b**) Degradation kinetic plot, ln(*C*_0_/*C_t_*) vs. irradiation time for MB (black points) and NB (red points).

Figure 8a shows the percentage degradation of dyes as a function of the irradiation time. The grown photocatalyst shows higher decolorization efficiency for NB as compared to MB. The difference in degradation efficiency of both dyes may be due to the different molecular structures of MB and NB. The percentage degradation of MB and NB is 95% and 97%, respectively, in 220 min under the sunlight. The kinetic studies reveal that the photocatalytic performance of NZP can be modeled by a pseudo-first-order kinetic reaction.

(3) $$C_{t}=C_{0}e^{-kt}$$

(4) $$\ln\left(\frac{C_{0}}{C_{t}}\right)=kt$$

where *k* is the rate constant, *C*_0_ the is initial concentration and *C_t_* the is concentration at time *t*. The rate constant k is the slope of the curve obtained by plotting *ln(C*_0_*/C_t_)* vs. irradiation time *t* as reported with solid line in Figure 8b. The value of k obtained for prepared NZP against MB and NB were 0.012 min^−1^ and 0.016 min^−1^, respectively. On the other hand, the value of R^2^ of the fitting were 0.970 and 0.971 for MB and NB, respectively, which also confirms the good choice of the pseudo first order reaction. The comparison of photodegradation efficiency of different metal oxide nanocomposite materials against MB and NB reported in literature is listed in Table 2. The values indicate that the photodegradation of our composite NZP against NB represents one of the best results obtained in the literature for binary composites to our knowledge.

For comparison, the self-degradation under sunlight of MB and NB in water solution without any catalyst was studied, and the results are shown in the supporting information. Regarding the Methylene Blue, the self-degradation was 50% in 220 min (see Figure S2), while for the NB, the self-degradation was about 26% after 220 min, as shown in Figure S3.

#### 3.6.2. Degradation of Bentazon Herbicide

**Figure 9:**
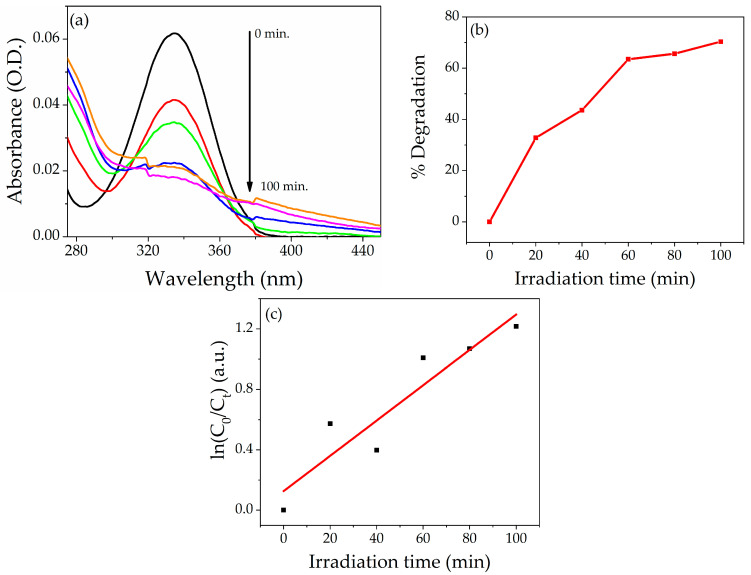
(**a**)Absorption spectra of Bentazon at 5 ppm in the presence of NZP. (**b**) Percentage degradation of NZP against Bentazon. (**c**) Photodegradation kinetic Plot, ln (*C*_0_/*C_t_*) vs. irradiation time.

The degradation efficiency of NZP was also investigated for BZ herbicides under UV light. The photodegradation of BZ is shown in Figure 9a. The maximum degradation was obtained after 100 min under UV light. The prepared photocatalyst shows 70% degradation of BZ after 100 min under UV light, as reported in Figure 9b. As in the case of dyes previously described the value of k of the curve slope for NZP for Bentazon was obtained by plotting ln(*C*_0_/*C_t_*) vs. irradiation time *t* Figure 9c. The calculated value of k obtained by the pseudo-first-order kinetic reaction is 0.011 min^−1^, and the value of R^2^ is 0.841. The comparison study of the photodegradation efficiency of different metal oxide nanocomposite materials reported in the literature for Bentazon herbicide is listed in Table 3.

Also in this case, the self-degradation of BZ was studied, and the results are shown in the supporting information in Figure S4. In the same time frame, the self-degradation reached about 38%. The presence of the catalyst is essential to boosting the degradation efficiency.

Concerning the reusability of the NZP catalyst, the efficiency in the three cycles is almost the same (~70%), as shown in Figure S5, underscoring that the composite can be reused several times.

### 3.7. Photodegradation Mechanism

When light strikes the NZP composite, electrons in the conduction band and holes in the valence band (*e*^−^_CB_ + *h*^+^_VB_) are generated. The oxidation and reduction processes take place at the surface of semiconductor photocatalysts. The expected photodegradation mechanism of the NiO-ZnO Photocatalyst can be summarized in four main steps:

Photo excitation:

$$\mathrm{NiO}-\mathrm{ZnO}+h\upsilon \;\to \;\mathrm{NiO}-\mathrm{ZnO}\;e_{CB}^{-}+{\;h}_{VB}^{+}$$

Oxygen ion absorption:

$$\mathrm{NiO}-\mathrm{ZnO}\;{(e}_{CB}^{-})+O_{2}\;\to \;\mathrm{NiO}-\mathrm{ZnO}+*O_{2}^{-}$$

Ionization of water:

H_2_O → H^+^ + *OH

*O_2_ + H^+^ → *HO_2_

Protonation of superoxide:

$$\mathrm{NiO}-\mathrm{ZnO}\;(e_{CB}^{-})+*{\mathrm{HO}}_{2}+H^{+}\;\to \;H_{2}O_{2}$$

$$\mathrm{NiO}-\mathrm{ZnO}\;{\;(h}_{VB}^{+})+\mathrm{dye}\;\mathrm{or}\;\mathrm{herbicide}\;\to \;\mathrm{degradation}\;\mathrm{products}$$

**Figure 10:**
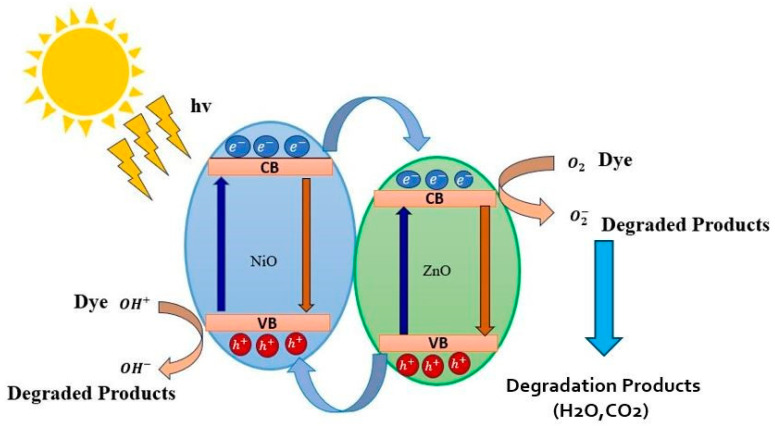
The schematic representation of photocatalytic mechanism for dyes in the presence of NZP.

Briefly, when light is irradiated on the NiO-ZnO photocatalyst, electron-hole pairs $\left(e_{CB}^{-}+{\;h}_{VB}^{+}\right)$ are generated. These photogenerated electrons react with oxygen molecules to form superoxide anion (*O_2_^−^) radicals that are less toxic, while the hole reacts with hydroxyl ions to form reactive hydroxyl (*OH) radicals. These excited radicals reduce the dye and herbicide molecules, while holes oxidize the pollutants directly and cause degradation. The combination of NiO-ZnO is able to create more dynamic catalytic centers, which assist in photodegradation [56]. Figure 10 represents the schematic diagram of the action of the NiO-ZnO photocatalyst.

## 4. Conclusions

In this study, pristine NiO, ZnO nanoparticles, and NiO-ZnO photocatalyst were synthesized and characterized for the degradation of organic pollutants. The NZP showed a smaller band gap energy (2.6 eV) compared with the pure NiO and ZiO components; consequently, this composite has a light absorption range from UV to natural light. The photocatalytic activity was investigated against MB, NB, and BZ; the degradation efficiency for dyes was 95%, 97% under sunlight, and 70% for herbicides under UV light, respectively. The photocatalyst has a recyclability of up to three cycles towards BZ without losing efficiency. Hence, this photocatalyst has great potential applications for wastewater treatment, the improvement of water quality discharge from textiles or other industries, and safeguarding the health of the ecological environment.

## Figures and Tables

**Table 1:** The structural parameters of NiO, ZnO and NiO-ZnO nanocomposite.

Oxides	*a* (A)	*c* (A)	*c/a*	Volume (Å^3^)	Micro Strain ε (×10^−4^)	d-Spacing (Å)	Dislocation Density (×10^−3^ nm^−2^)
Individual							
NiO	4.184	-	1	72.748	2.025	1.686	3.139
ZnO	3.243	5.209	1.603	47.609	9.838	2.041	0.746
In NZP							
NiO	4.194	-	1	73.786	12.885	1.694	1.337
ZnO	3.251	5.219	1.605	47.687	12.019	1.994	1.130

**Table 2:** The comparison of photodegradation efficiency of different metal oxide nanocomposite materials against MB and NB.

Photocatalyst	Dyes	Source	Degradation Efficiency (%)	Ref.
ZnO-MgO	MB	Sunlight	89	[30]
ZnO-CdO	-	-	97	[28]
WO_3_-ZnO	-	-	90	[52]
NiO-ZnO	-	-	95	present work
CuO-SiO_2_	NB	UV-Visible	90	[53]
FeMnO_3_	-	Sunlight	95	[54]
CuFe_2_O_4_	-	Hg lamp	93	[55]
NiO-ZnO	-	Sunlight	97	present work

**Table 3:** The comparison study of photodegradation efficiency of different metal oxide nanocomposite materials against Bentazon herbicide.

Photocatalyst	Source	Irradiation Time (min)	Degradation Efficiency (%)	Ref.
Fe_2_O_3_-TiO_2_	UV-Visible lamp	120	51	[1]
N–TiO_2_–PMAA-g-PVDF/PAN	UV light	180	90	[6]
NiO-ZnO	UV light	100	70	Present work

## Data Availability

Data are contained within the article and supplementary materials.

## Supplementary Materials

The following supporting information can be downloaded at: https://www.mdpi.com/article/10.3390/nano14050470/s1, Figure S1. Degradation efficiency of BZ as a function of pH value; Figure S2. Absorption spectra of MB at 5 ppm in the absence of catalyst under sunlight irradiation; Figure S3. Absorption spectra of NB at 5 ppm in the absence of catalyst under sunlight irradiation; Figure S4. Absorption spectra of BZ at 5 ppm in the absence of catalyst under UV lamp; Figure S5: Percentage degradation of BZ under three different cycles.
