# Supplementary material for: Photocatalytic Degradation of Organic Pollutants—Nile Blue, Methylene Blue, and Bentazon Herbicide—Using NiO-ZnO Nanocomposite
Source: Nanomaterials (Basel). 2024 Mar 5;14(5):470. doi: 10.3390/nano14050470 (PMC10935274; doi:10.3390/nano14050470)
Supplement: Supplementary file 1 [file nanomaterials-14-00470-s001.zip › nanomaterials-2878955-supplementary.pdf]

Supporting Information

Photocatalytic Degradation of Organic Pollutants—Nile Blue, Methylene Blue, and Bentazon Herbicide—Using NiO-ZnO Nanocomposite

Sadaf Yasmeen [1], Luca Burratti [2,*], Leonardo Duranti [3], Emanuela Sgreccia [1] and Paolo Prosposito [1]

[1] Industrial Engineering Department, University of Rome Tor Vergata, Via del Politecnico 1, 00133 Rome, Italy; sadaf.yasmeen@students.uniroma2.eu (S.Y.); emanuela.sgreccia@uniroma2.it (E.S.); paolo.prosposito@uniroma2.it (P.P.)
[2] Department of Sciences, University of Roma Tre, Via della Vasca Navale 79, 00146 Rome, Italy
[3] Department of Chemical Science and Technologies, University of Rome Tor Vergata, Via della Ricerca Scientifica 1, 00133 Rome, Italy; leonardo.duranti@uniroma2.it
* Correspondence: luca.burratti@uniroma3.it

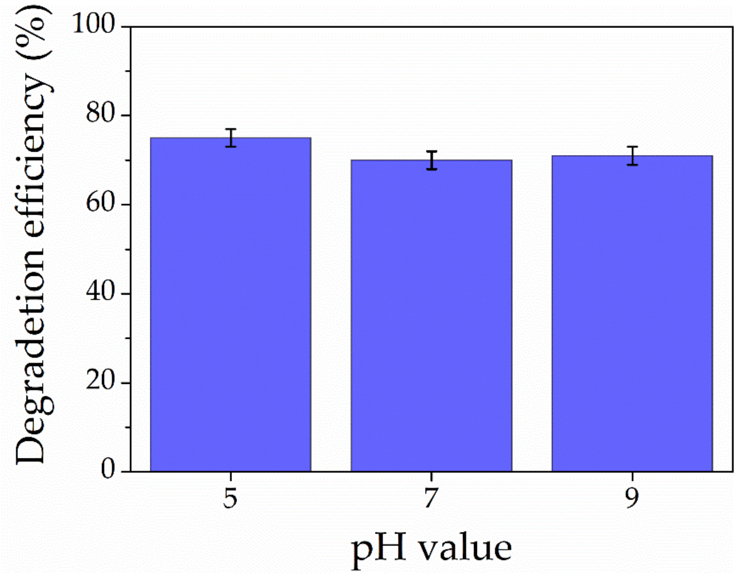

Figure S1. Degradation efficiency of BZ as a function of pH value.

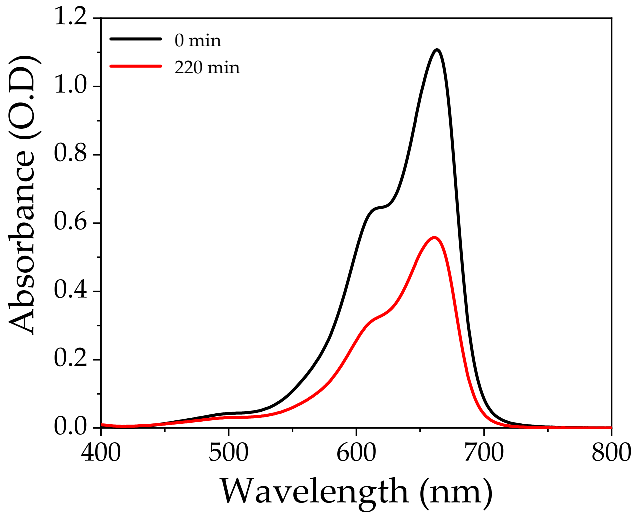

Figure S2. Absorption spectra of MB at 5ppm in the absence of catalyst under sunlight irradiation.

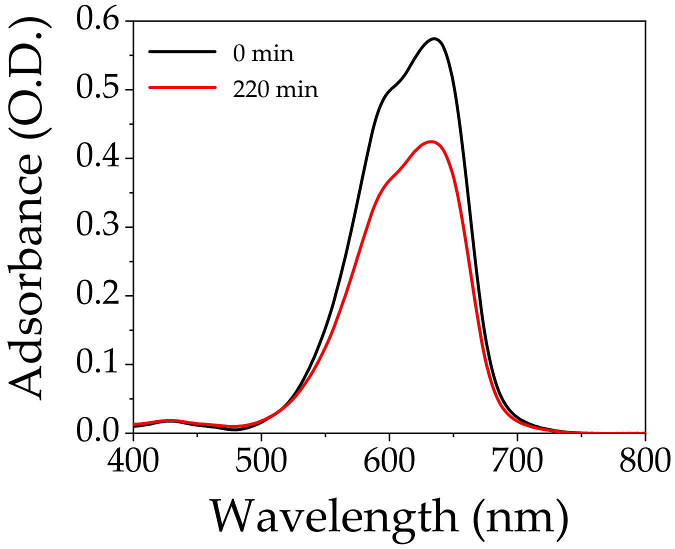

Figure S3. Absorption spectra of NB at 5ppm in the absence of catalyst under sunlight irradiation.

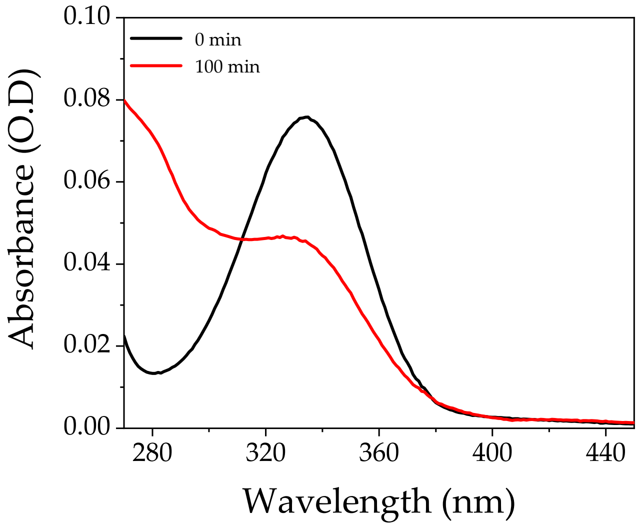

Figure S4. Absorption spectra of BZ at 5ppm in the absence of catalyst under UV lamp.

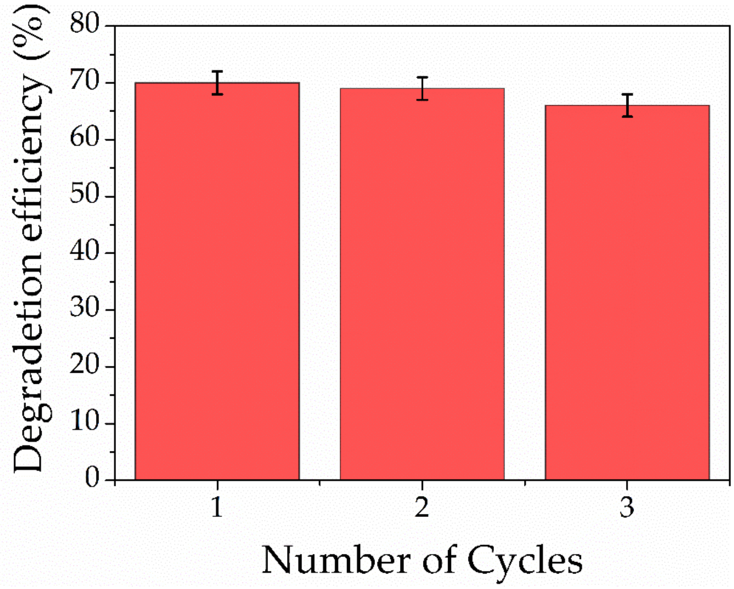

Figure S5. Percentage degradation of BZ under three different cycles.
